# Tunable Crosslinked Polyvinyl Alcohol/Polyethylene Glycol (cPVA/PEG) Nanofiber Membranes with Enhanced Mechanical and Hydrophilic Balance

**DOI:** 10.3390/molecules30183750

**Published:** 2025-09-15

**Authors:** Yawen Chang, Zijia Wang, Fujuan Liu

**Affiliations:** National Engineering Laboratory for Modern Silk, College of Textile and Clothing Engineering, Soochow University, 199 Ren-Ai Road, Suzhou 215123, China; 20225215004@stu.suda.edu.cn (Y.C.); 20245215006@stu.suda.edu.cn (Z.W.)

**Keywords:** crosslinked PVA, electrospinning, hydrophilic, nanofiber membranes, mechanical

## Abstract

In recent years, membrane separation technology has undergone continuous advancements. Microfiltration (MF) membranes, as an important type, are usually prepared by electrospinning—a simple and efficient method. This study reports the development of crosslinked polyvinyl alcohol/polyethylene glycol (cPVA/PEG) nanofiber membranes through a combination of electrospinning and chemical crosslinking, investigating the effects of different crosslinking concentrations on the membrane morphology, surface wettability, and tensile properties. Comprehensive characterization was carried out by using scanning electron microscopy (SEM), a Fourier-transform infrared spectrometer (FTIR), an X-ray diffractometer (XRD), a thermogravimetric (TG) analyzer, differential scanning calorimetry (DSC), a contact angle tester, a universal testing machine, etc. The results showed that at the crosslinking concentration of 15%, the cPVA/PEG fiber membrane achieved a breaking stress of 29.07 ± 2.60 MPa, a breaking strain of 77.60 ± 6.02%, and a porosity exceeding 43%. SEM, FTIR, XRD, TG, and DSC analyses collectively confirmed the occurrence of chemical crosslinking within the membrane structure. The cPVA/PEG-15 membrane exhibited no observable shrinkage or curling upon water contact, combined with excellent hydrophilicity and lipophilicity in the air. These properties indicate that the membrane can serve as a novel functional membrane substrate (e.g., as hydrophilic separation layers) and is expected to play an important role in fields such as seawater desalination and wastewater treatment, demonstrating significant application potential.

## 1. Introduction

In the early 20th century, a series of new breakthroughs and developments emerged in the basic theoretical framework of membrane separation. For example, the equilibrium phenomenon in charge transfer was studied in depth, and the relationship between osmotic pressure behaviors and thermodynamic properties was gradually clarified [1]. In 1950, Jada et al. [2] successfully prepared ion-exchange membranes for the first time, which promoted the rapid development of subsequent electrodialysis (ED) technology. The milestone came in 1965 when Loeb et al. [3] invented asymmetric reverse osmosis (RO) membranes, enabling the large-scale industrial application of membrane separation. From the 1970s to the 1990s, significant research progress was achieved in subfields such as ultrafiltration (UF) [4], gas separation (GS) [5], and osmotic evaporation (PV) [6]. With the continuous technological advancements, membrane separation has been increasingly deepened and expanded into specialized domains in recent years. Through combining with many emerging technologies, it has driven more profound applications and developments across industries including chemical engineering, environmental protection, pharmaceuticals, and food science [7,8,9].

Membrane separation technology employs semi-permeable membranes as the filtration layers to achieve the selective separation of gas or liquid mixtures with distinct components. The permeation process is accelerated by applying external forces, electric field forces, etc. This technology has significant advantages such as operational simplicity, low energy consumption, high separation efficiency, cost-effectiveness, and easy scalability [10,11]. Among membrane processes, microfiltration (MF) features a pore size distribution of 0.1–1 μm, primarily used for removing micrometer-scale suspended particles, bacteria, and microorganisms, with extensive applications in wastewater treatment and water purification [12]. Gonçalves et al. [13] prepared polyacrylonitrile nanofiber membranes by electrospinning. The membranes exhibited an average diameter of 580 nm, 79.5% porosity, and a pure water flow rate of 19,500 L h^−1^ m^−2^, effectively retaining 1.75 μm particles and demonstrating capability for efficient suspension particle separation. Rashad et al. [14] developed a single-piece ceramic microfiltration membrane with mullite crystal interchain networks, synthesized using low-cost clay and Al_2_O_3_ as raw materials. This membrane reached 64% porosity, a 0.3 μm median pore diameter, 43 MPa flexural strength, and 1031 L m^−2^ h^−1^ bar^−1^ pure water permeability. Compared with traditional asymmetric ceramic membranes, it offers an economical and efficient method for separating oil-in-water emulsions.

MF membranes typically exhibit hydrophilic surfaces. Common hydrophilic polymers used for preparing surface hydrophilic membranes include polyacrylonitrile (PAN) [15], cellulose [16,17], polyvinyl alcohol (PVA) [18], polyethylene glycol (PEG) [19], etc. The hydrophilic functional groups (such as -OH, -COOH, and -NH_2_) of hydrophilic polymers endow the resulting fiber membranes with surface hydrophilicity [20,21]. The fluctuation of the polymer chains can accelerate the spread of water droplets on the fiber membrane surface, thereby promoting membrane permeability [22]. However, when pure hydrophilic polymer membranes are exposed to water for extended periods, complete water penetration occurs, resulting in severe deformation and affecting their subsequent applications.

PVA is a water-soluble polymer with high biodegradability, elasticity, and low cost [23], offering extensive application scenarios. Under certain conditions, the hydrophilic groups (-OH) in PVA react with water, causing the molecular chains to split. This inherent limitation precludes PVA from being used as the sole raw material for hydrophilic membranes. However, PVA can form water-insoluble crosslinked polymers via crosslinking agents such as glutaraldehyde, formaldehyde, and tannic acid [18,24,25], which significantly improve mechanical properties. Meanwhile, the hydrogen bonds within PVA enable its combination with other polymers (such as PEG, PVP) [26,27] to form composite polymers that inherit most physicochemical properties [28], opening up more application possibilities. do Nascimento et al. [29] studied PVA membranes crosslinked with citric acid, succinic acid, and their mixtures. The results showed that the crosslinked membranes had uniform surface roughness, superior crosslinking efficiency, and enhanced adhesion and mechanical properties. Zhang et al. [30] prepared crosslinked PVA/PAN composite fiber membranes for wastewater treatment. Using pyrodianhydride (PMDA) as a crosslinker, the composite membranes were fabricated by a coating method. When tested with 35,000 ppm NaCl aqueous solution, the membrane crosslinked at 100 °C for 2 h achieved the highest NaCl removal rate (99.98%) with a water flux of 32.26 L m^−2^ h^−1^.

In this study, PVA served as the substrate, PEG was added as the adhesive modifier to enhance the interfiber adhesion, and GA acted as the crosslinking agent. Through electrospinning technology, cPVA/PEG nanofiber membranes that met the practical application requirements were prepared by varying the crosslinking concentration (0%, 3%, 9%, 15%, 30%, 50%). The effects of crosslinking concentrations on the scanning electron microscopy (SEM) morphology, Fourier-transform infrared spectroscopy (FTIR) characteristics, thermogravimetric (TG) analysis, differential scanning calorimetry (DSC), contact angle, and mechanical properties of fiber membranes were systematically investigated. The aim was to develop functional membrane substrates suitable for hydrophilic separation layers, thereby demonstrating their significant application value in seawater desalination.

## 2. Results and Discussion

### 2.1. Morphology of Nanofiber Membranes

**Figure 1:**
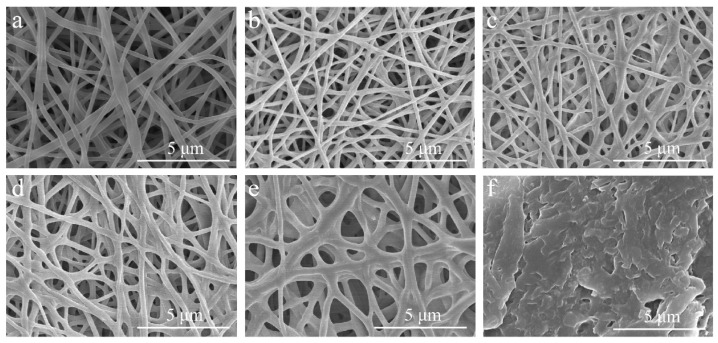
Surface magnification SEM images of PVA/PEG and cPVA/PEG fiber membranes at different crosslinking concentrations: (**a**) PVA/PEG, (**b**) cPVA/PEG-3, (**c**) cPVA/PEG-9, (**d**) cPVA/PEG-15, (**e**) cPVA/PEG-30, (**f**) cPVA/PEG-50.

The surface SEM images of the PVA/PEG and cPVA/PEG fiber membranes with different crosslinking concentrations are shown in Figure 1. Due to the bonding effect of PEG, interfiber adhesion and entanglement phenomena occurred in the PVA/PEG fiber membranes (Figure 1a). As the crosslinking concentration increased to 15%, the interfiber adhesion gradually intensified, accompanied by an increase in the fiber diameter and the formation of sheet-like adhesion structures (Figure 1b–d). Further increasing crosslinking concentration, the proportion of single fibers in the cPVA/PEG-30 fiber membrane decreased, and the fibers adhered to form a lotus sheet-like structure (Figure 1e). The fibrous structure could no longer be observed in the cPVA/PEG-50 fibrous membrane (Figure 1f), with excessive interfiber adhesion, indicating that the crosslinking concentration was too high, which may have an adverse effect on the membrane porosity.

### 2.2. Infrared Analysis

**Figure 2:**
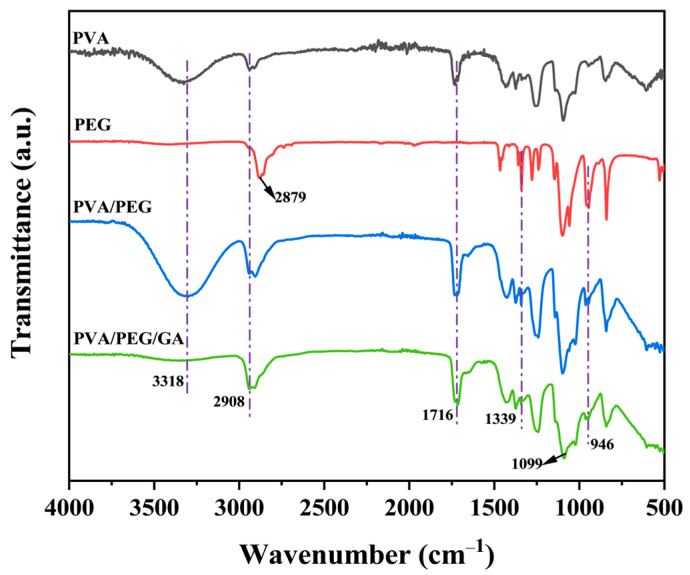
Infrared spectra of PVA, PEG, PVA/PEG, and cPVA/PEG-15 fiber membranes.

Figure 2 presents the infrared spectra of PVA, PEG, PVA/PEG, and cPVA/PEG-15 fiber membranes, which were used to analyze the chemical groups on the membrane surfaces and verify the successful crosslinking of PVA and GA. For the PVA fiber membrane, the O-H stretching vibration peak appeared at 3318 cm^−1^, and the strong absorption peak at 1716 cm^−1^ was related to the asymmetric stretching vibration of C=O [31]. Observing the FTIR spectra of pure PEG, it could be known that the main characteristic peaks include a -CH_2_ stretching vibration absorption peak at 2879 cm^−1^, as well as the bending/stretching vibration absorption peaks of C-O-C corresponding to 1099 cm^−1^ and 946 cm^−1^, respectively [32]. FTIR analysis indicated that the PVA/PEG fiber membrane had corresponding characteristic peaks of both components, confirming physical blending and retention of their respective chemical properties. Compared with PVA, the cPVA/PEG-15 fiber membrane displayed a significant reduction in the intensity of the O-H stretching vibration characteristic peak at 3318 cm^−1^. This is attributed to the reaction of hydroxyl groups in PVA with the aldehyde groups in GA to form acetal or semi-acetal groups [33]. The vibration of the acetal group was evidenced by peak broadening between 1000 and 1140 cm^−1^ [34]. Meanwhile, PEG-related groups remained in the cPVA/PEG-15 fiber membrane, indicating the physical retention of PEG and preservation of its chemical properties. The characteristic peaks between 2730 and 2860 cm^−1^ may originate from the incompletely reacted GA in the fibrous membrane [35], but overlapping with the characteristic peaks of PEG hinders definitive assignment. Overall, the infrared spectroscopy results confirm that the crosslinking of PVA and GA in the cPVA/PEG-15 fiber membrane was successful, which is consistent with the literature [36].

### 2.3. XRD Analysis

**Figure 3:**
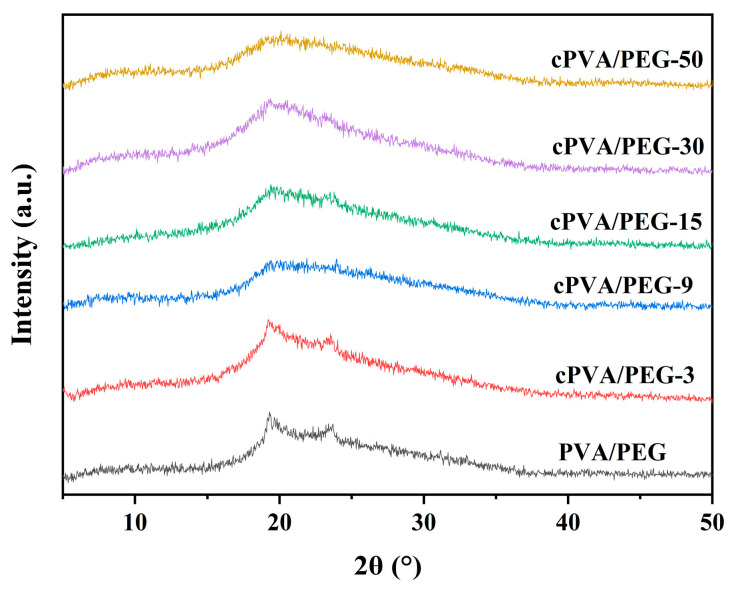
XRD spectra of PVA/PEG and cPVA/PEG nanofiber membranes.

The XRD spectra of PVA/PEG and cPVA/PEG fiber membranes are shown in Figure 3. XRD can be used to not only determine the properties of compounds but also characterize the structural parameters of polymer crystals. As shown, the PVA/PEG nanofiber membrane revealed a wide diffraction peak at around 19°, corresponding to the characteristic peak of PVA [37]. The broad signal centered at 24° was attributed to the characteristic peak of PEG [32]. With the increase in crosslinked concentration, the characteristic peak intensity of PVA in cPVA/PEG nanofiber membranes showed a weakening trend. This is due to the chemical crosslinking between PVA and GA, which disrupts the crystalline structure of PVA, or potentially to the influence of unreacted GA on the signal intensity. As the GA volume fraction increased, the diffraction peaks of the fiber membrane broadened, indicating a decrease in crystallite size, and even the formation of an amorphous state. These structural changes are correlated with the enhanced tensile strength of the membranes, consistent with the results of the mechanical properties discussed later.

### 2.4. Thermal Analysis

**Figure 4:**
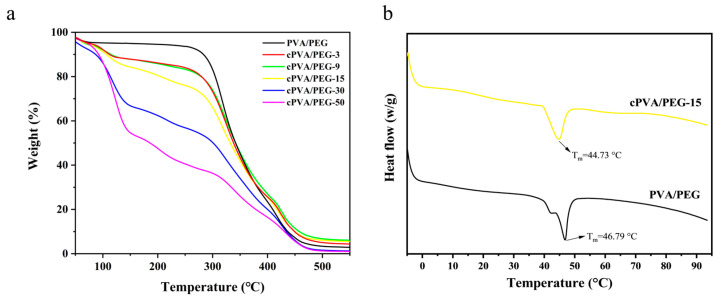
(**a**) TG curves of PVA/PEG and cPVA/PEG nanofiber membranes, (**b**) DSC profiles of PVA/PEG and cPVA/PEG-15 nanofiber membranes (heating rate = 10 °C/min, exothermic up).

The TG curves of PVA/PEG and cPVA/PEG nanofiber membranes are exhibited in Figure 4a. As can be seen from the figure, the thermal decomposition process of the PVA/PEG fiber membrane presented three stages. The first stage (0–100 °C) corresponded to the evaporation of residual moisture on the membrane surface. The second stage (100–300 °C) demonstrated a negligible weight change. The third stage began at 300 °C, and the molecular chains of PVA and PEG underwent extensive breaks, resulting in rapid weight loss and stabilizing by 450 °C [38,39]. At the same time, the thermal decomposition of cPVA/PEG nanofiber membranes also comprised three stages. The first stage was consistent with that of PVA/PEG nanofiber membranes. In the second stage (100–300 °C), the mass loss gradually increased with the rise in crosslinking concentration, indicating that the mass loss in this stage originated from the decomposition of the oxygen-containing functional groups in unreacted GA remaining in the membrane. The third stage (after 300 °C) mainly involved the cleavage of cPVA and PEG molecular chains, further decomposition of GA-derived intermediates, and partial carbonization of decomposition products. The comprehensive analysis illustrated that the content of uncrosslinked GA was relatively low inside the fiber membranes with crosslinking concentrations of 3%, 9%, and 15%. XRD analysis showed that as the volume fraction of GA increases, the interior of the fiber membranes gradually turned into an amorphous structure. However, the amorphous state, lacking a distinct melting point and featuring an unstable internal structure, was prone to lead to poor thermal stability, consistent with the TG results.

In addition, the DSC profiles of PVA/PEG and cPVA/PEG-15 nanofiber membranes within the temperature range of −5~95 °C are presented in Figure 4b. The melting point (T_m_) of the crosslinked cPVA/PEG-15 fiber membrane was lower than that of the PVA/PEG fiber membrane, and its melting peak was broader—indicating lower crystallinity or a wider crystallite size distribution. Furthermore, the melting enthalpy (ΔH) of the PVA/PEG fiber membrane was 10.95 J/g, higher than the 8.53 J/g of the cPVA/PEG-15 fiber membrane. This result suggests that the former possesses higher crystallinity and a more ordered structure, thus requiring more energy absorption during melting. In contrast, the cPVA/PEG-15 fiber membrane exhibits reduced crystallinity due to crosslinking restricting molecular chain alignment. This finding is consistent with the results from the XRD analysis.

### 2.5. Porosity

Table 1 provides the porosity of PVA/PEG and cPVA/PEG nanofiber membranes at varying crosslinking concentrations. As the crosslinking concentration increased from 3% to 50%, the porosity of the cPVA/PEG fiber membrane continuously decreased from 48.23 ± 5.72% to 15.02 ± 2.81%. Compared with PVA/PEG, the porosities of the cPVA/PEG membranes with crosslinking concentrations of 3%, 9%, and 15% showed slight reduction, but the differences were not significant, with all values remaining above 43%. Conversely, further increases in the crosslinking concentration led to a more pronounced reduction in porosity, which may be attributed to the gradual increase in the interfiber adhesion structure (specifically in Figure 1).

### 2.6. Contraction upon Contact with Water

**Figure 5:**
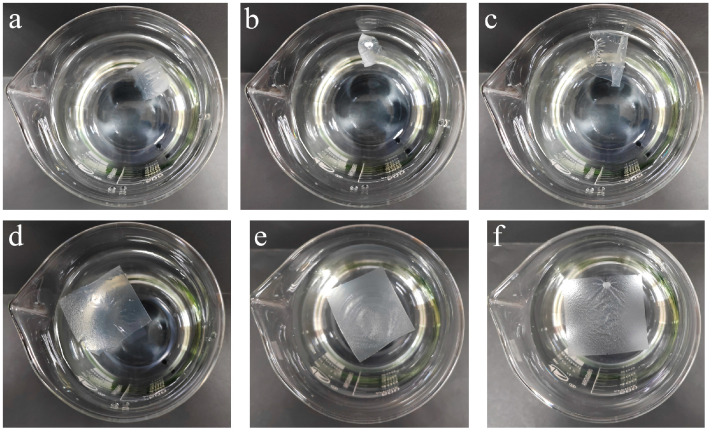
Contraction diagrams in water of PVA/PEG and cPVA/PEG fiber membranes at different crosslinking concentrations: (**a**) PVA/PEG, (**b**) cPVA/PEG-3, (**c**) cPVA/PEG-9, (**d**) cPVA/PEG-15, (**e**) cPVA/PEG-30, and (**f**) cPVA/PEG-50.

To meet the requirements for subsequent recyclable applications, crosslinked fiber membranes need to have the characteristics of water resistance (i.e., no dissolution or shrinkage when exposed to water). Figure 5 demonstrates the water shrinkage behavior of PVA/PEG membranes and cPVA/PEG nanofiber membranes at different crosslinking concentrations. When the crosslinking concentration was below 9% (0–9%), the fiber membranes contracted and curled rapidly upon water contact (Figure 5a–c). This is attributed to insufficient crosslinking, causing PVA and GA to fail to react fully to form water-insoluble crosslinked compounds. Therefore, the water-soluble property of the PVA/PEG matrix is fundamentally unchanged. With the increase in crosslinking concentration, the fiber membrane basically no longer shrank or curled after water contact (Figure 5d–f). And no dissolution occurred over time. This indicates that complete crosslinking was achieved when the crosslinking concentration reached 15% or higher.

### 2.7. Surface Water Wettability

**Figure 6:**
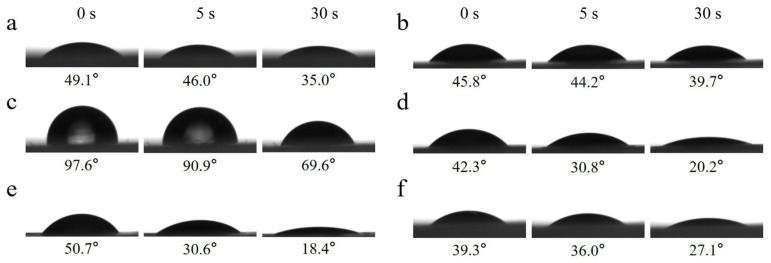
Dynamic water contact angle images of PVA/PEG and cPVA/PEG fiber membranes at different crosslinking concentrations. (**a**) PVA/PEG, (**b**) cPVA/PEG-3, (**c**) cPVA/PEG-9, (**d**) cPVA/PEG-15, (**e**) cPVA/PEG-30, and (**f**) cPVA/PEG-50.

Figure 6 shows the dynamic water contact angles measured for PVA/PEG and cPVA/PEG membranes at different crosslinking concentrations in the air. Within 30 s, the dynamic water contact angle of PVA/PEG decreased from 49.1° to 35.0°, while that of cPVA/PEG also exhibited a downward trend. Among them, cPVA/PEG-3 presented the smallest change in the water contact angle on the fiber membrane surface within 30 s (a decrease of 6.1°), whereas cPVA/PEG-30 showed the greatest change (a decrease of 32.3°). Except for the initial water contact angle of the cPVA/PEG-9 fiber membrane being slightly greater than 90°, indicating hydrophobicity, the initial water contact angles of the fiber membranes at other crosslinking concentrations were all much less than 90°, showing hydrophilicity. In conclusion, when the crosslinking concentration was 15% or 30%, the surface hydrophilicity of the crosslinked fiber membrane was relatively excellent.

### 2.8. Surface Oil Wettability

**Figure 7:**
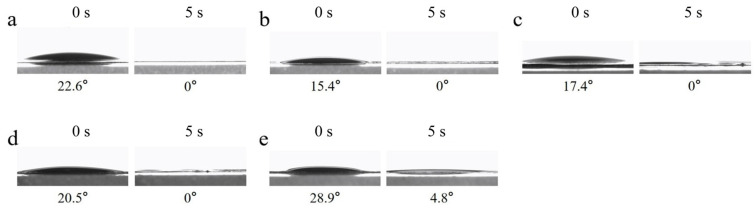
Dynamic oil contact angle images of cPVA/PEG fiber membranes at different crosslinking concentrations: (**a**) cPVA/PEG-3, (**b**) cPVA/PEG-9, (**c**) cPVA/PEG-15, (**d**) cPVA/PEG-30, and (**e**) cPVA/PEG-50.

The wettability of the fiber membrane surfaces to oil in air is another critical criterion for evaluating surface wettability. The dynamic oil contact angle images of cPVA/PEG nanofiber membranes under different crosslinking concentrations are shown in Figure 7. The cPVA/PEG nanofiber membrane presented the initial oil contact angle ranging from 15.4° (cPVA/PEG-9) to 28.9° (cPVA/PEG-50). All the oil contact angles were less than 90°, indicating the universal lipophilicity of the fiber membrane surfaces. Except for the cPVA/PEG-50, the oil on the surface of other crosslinking membranes was completely absorbed after 5 s. In short, the surface of the fiber membrane was lipophilic in the air and possessed good oil absorption performance.

### 2.9. Tensile Properties

**Figure 8:**
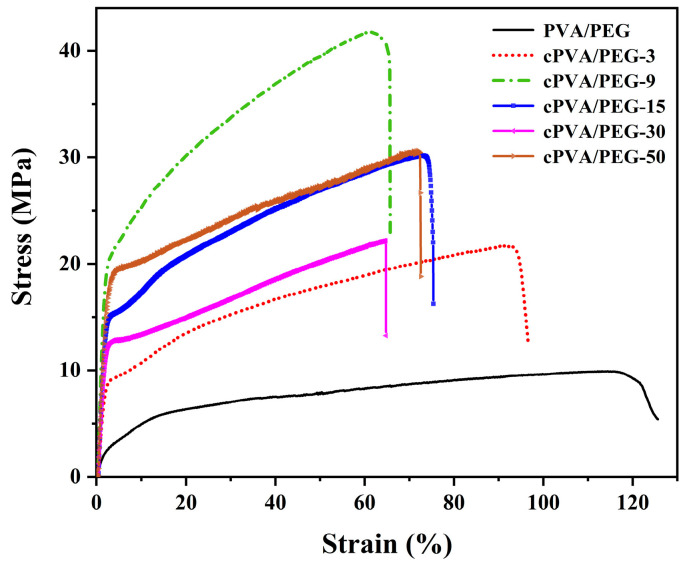
Tensile properties of PVA/PEG and cPVA/PEG nanofiber membranes.

The stress/strain curves of PVA/PEG and cPVA/PEG membranes at different crosslinking concentrations are presented in Figure 8, and the corresponding breaking stress and strain values are summarized in Table 2. The addition of PEG significantly enhanced the breaking strain of the PVA/PEG fiber membrane to 115.44%, attributable to the certain adhesiveness of PEG itself, which promoted interfiber bonding (Figure 1a). At a 3% crosslinking concentration, the breaking stress of the fiber membrane increased markedly to 21.91 MPa, but the breaking strain decreased to 90.57%, likely due to the occurrence of chemical crosslinking inside the membrane structure. The breaking stress of the cPVA/PEG-9 membrane reached the maximum value of 39.21 MPa, accompanied by the lowest fracture strain of 57.56%. This was attributed to increased GA in the spinning solution, enabling more acetal reactions with PVA to form a denser crosslinked network that strengthened interfiber interactions. When the crosslinking concentration was further increased to 30%, the breaking strain of the fiber membrane showed a trend of first increasing (77.60%) and then decreasing (63.61%), while the breaking stress declined continuously to 22.49 MPa. At 50% of the crosslinking concentration, both the breaking strain and stress increased to some extent. To summarize, the cPVA/PEG-15 and cPVA/PEG-50 fiber membranes demonstrated relatively superior mechanical properties.

Although electrospun nanofiber membranes are well recognized for their high porosity and tunable morphology, their mechanical strength is often insufficient [40]—limiting their practical applications. Table 3 provides a comparison of the mechanical properties of various electrospun nanofiber membranes for water treatment, as reported in the recent literature. It can be observed that the breaking stress of these membranes is generally low, mostly in the range of 1~10 MPa. In contrast, the cPVA/PEG-15 membrane prepared in this study via electrospinning coupled with GA crosslinking demonstrates exceptional mechanical properties, with a breaking stress of 29.07 MPa. This enhancement could be attributed to the unique crosslinked network structure formed between GA and PVA, which effectively improved the interfiber bonding and load-transfer capacity, thereby improving the mechanical strength of the membrane.

## 3. Materials and Methods

### 3.1. Materials

Polyvinyl alcohol 1788 (PVA, Mw = 85,000 g/mol) and glutaraldehyde (GA, 50% in H_2_O) were provided by Shanghai Aladdin Biochemical Technology Co., Ltd. (Shanghai, China); polyethylene glycol (PEG, Mw = 4000 g/mol) was purchased from Shanghai yuanye Bio-Technology Co., Ltd. (Shanghai, China); anhydrous ethanol and n-hexane were obtained from Jiangsu Qiangsheng Functional Chemistry Co., Ltd. (Suzhou, China); N-butanol was supplied by Sinopharm Chemical Reagent Co., Ltd. (Shanghai, China). All reagents are analytically pure.

### 3.2. Preparation of Crosslinked PVA/PEG Hydrophilic Nanofiber Membranes

**Figure 9:**
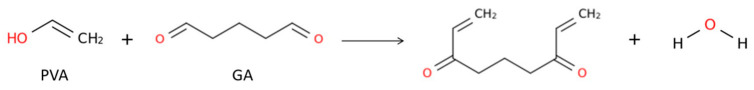
Diagram of the chemical reaction between PVA and GA.

A certain amount of PVA and PEG powders was dissolved in deionized water and stirred in a 70 °C constant temperature water bath for 6 h to prepare a 10 wt.% PVA/PEG spinning solution, in which the mass ratio of PVA to PEG was 9:1. The solution was then kept at room temperature for 3 h to remove the bubbles. Crosslinking between PVA and GA occurs through an acetal reaction, that is, the hydroxyl groups on the PVA molecular chain react with the aldehyde group in GA to form an acetal bond [48]. The process of the chemical reaction to form crosslinked polymers between PVA and GA is shown in Figure 9.

The obtained PVA/PEG spinning solution was mixed with the GA solution at a specific volume ratio (100/0, 97/3, 91/9, 85/15, 70/30, 50/50) under stirring to prepare the crosslinked PVA/PEG spinning solutions. These solutions were loaded into syringes for electrospinning. The parameters were set as follows: the distance between the needle and the drum receiving device of 12 cm, the spinning voltage of 20 kV, the spinning speed of 1 mL/h, and the drum rotation speed of 400 rpm. In addition, the indoor temperature was maintained at 22 °C and the relative humidity (RH) at 50% during the preparation process. After spinning, the as-obtained fiber membranes were dried in an oven at 60 °C for 3 h to remove the residual solvent. And then, they were noted as PVA/PEG, cPVA/PEG-3, cPVA/PEG-9, cPVA/PEG-15, cPVA/PEG-30, and cPVA/PEG-50, respectively.

### 3.3. Characterization

The surface morphology of the nanofiber membranes was observed using a cold field emission scanning electron microscope (FE-SEM, S-4800, Hitachi, Tokyo, Japan). The functional groups and chemical structure were analyzed via a Fourier-transform infrared spectrometer (FTIR, Nicolet5700 is5, Thermo Fisher Scientific, Waltham, MA, USA) and by X-ray diffractometer (XRD, D8 Advance, Bruker, Billerica, MA, USA). Thermogravimetric (TG, Diamond 5700, PerkinElmer, Waltham, MA, USA) analysis was performed to investigate structural changes inside the fiber membranes. Differential scanning calorimetry (DSC 250, TA Instruments, New Castle, DE, USA) was employed to characterize the thermal behavior of the PVA/PEG fiber membrane and cPVA/PEG-15 fiber membrane. The tensile properties were characterized via a universal testing machine (Instron 5967, Instron, Norwood, MA, USA), and the porosity was determined by the liquid adsorption method. Finally, the contact angle changes on the fiber membrane surface were also recorded using a contact angle tester (OCA40, DataPhysics Instruments, Filderstadt, Germany).

## 4. Conclusions

In conclusion, cPVA/PEG nanofiber membranes were successfully prepared by electrospinning combined with chemical crosslinking, which exhibited excellent mechanical and hydrophilic properties. Compared to PVA/PEG fiber membranes, the crosslinked counterparts had enhanced adhesive structures among the fibers. FTIR, XRD, TG, and DSC collectively confirmed that the acetal reaction occurred between PVA and GA inside the fiber membrane matrix. The tensile test results showed that the breaking stress of the crosslinked membrane had been significantly increased. Notably, the cPVA/PEG-15 membrane achieved a breaking stress of 29.07 ± 2.60 MPa and a breaking strain of 77.60 ± 6.02%, with a porosity exceeding 43%. Upon water contact, no observable shrinkage or curling phenomenon was found in the cPVA/PEG-15 membrane, which maintained excellent hydrophilicity and lipophilicity in the air. These attributes highlight its broad application prospects in fields such as seawater desalination, wastewater treatment, and related environmental and industrial sectors.

## Figures and Tables

**Table 1:** The porosity of PVA/PEG and cPVA/PEG nanofiber membranes.

Samples	Porosity (%)
PVA/PEG	50.69 ± 3.79
cPVA/PEG-3	48.23 ± 5.72
cPVA/PEG-9	45.12 ± 5.06
cPVA/PEG-15	43.89 ± 4.22
cPVA/PEG-30	31.95 ± 1.21
cPVA/PEG-50	15.02 ± 2.81

**Table 2:** The breaking stress and strain of PVA/PEG and cPVA/PEG nanofiber membranes.

Samples	Breaking Stress (MPa)	Breaking Strain (%)
PVA/PEG	8.79 ± 0.93	115.44 ± 30.58
cPVA/PEG-3	21.91 ± 0.61	90.57 ± 1.16
cPVA/PEG-9	39.21 ± 3.71	57.56 ± 6.11
cPVA/PEG-15	29.07 ± 2.60	77.60 ± 6.02
cPVA/PEG-30	22.49 ± 2.67	63.61 ± 7.70
cPVA/PEG-50	30.58 ± 1.66	69.91 ± 5.07

**Table 3:** Comparison of the mechanical properties of various electrospun nanofiber membranes for water treatment applications.

Materials	Preparation Methods	Breaking Stress (MPa)	Breaking Strain (%)	Origin	Manufacturer	References
PVC/TPU/PC electrospun nanofiber membranes	Electrospinning	3.5~10.3	41~56	Bonab, Iran	Yekrang et al.	[41]
Heat-treated PPSU ENM	Electrospinning combined with thermal treatment	4.1 ± 0.2	34.4 ± 4.5	Kongens Lyngby, Denmark	Wang et al.	[42]
PA-modified PAN nanofiber membranes	Electrospinning	1.65 ± 0.21	53.0 ± 10.1	Jiangmen, China	Li et al.	[43]
PDMS/PVDF membranes	Electrospinning	1.84	68.30	Beijing, China	Li et al.	[44]
PVDF-PVDF/PDA NFMs	Double-nozzle electrospinning	<2.052	<49.382	Jinan, China	Feng et al.	[45]
PLA fiber membrane with silica layer	Electrospinning	2.395	44.1	Yancheng, China	Gao et al.	[46]
SiO_2_@PEI-PAN/PVB nanofiber membrane	Coaxial electrospinning	2.4	13.3	Suzhou, China	Ma et al.	[47]
cPVA/PEG-15 membrane	Electrospinning	29.07 ± 2.60	77.60 ± 6.02	Suzhou, China	Chang et al.	This work

## Data Availability

The original contributions presented in this study are included in the article. Further enquiries can be directed to the corresponding author.
